# Bridging the Access Gap for Comprehensive Sickle Cell Disease Management Across Sub-Saharan Africa: Learnings for Other Global Health Interventions?

**DOI:** 10.5334/aogh.4132

**Published:** 2023-11-14

**Authors:** Lutz Hegemann, Vas Narasimhan, Kwaku Marfo, Patrick Kuma-Aboagye, Solomon Ofori-Acquah, Isaac Odame

**Affiliations:** 1 Global Health & Sustainability, Novartis International AG, Basel, Switzerland; 2 Novartis International AG, Basel, Switzerland; 3 Ghana Health Service, Headquarters PMB, Ministries, Accra, Ghana; 4 West African Genetic Medicine Center (WAGMC), University of Ghana, Accra, Ghana; 5 Sickle Cell Foundation of Ghana, Accra, Ghana; 6 University of Pittsburgh, Pittsburgh, PA, USA; 7 Division of Haematology/Oncology and Centre for Global Child Health, The Hospital for Sick Children (SickKids),Toronto, ON, Canada; 8 Temerty Faculty of Medicine, University of Toronto, ON, Canada

**Keywords:** Sickle Cell Disease, Sustainability, Public-private partnerships, Healthcare access gaps, Cross-sector collaborations, Global health burden

## Abstract

**Background:**

Sickle cell disease (SCD) is a major unresolved global health issue, with the highest disease burden in sub-Saharan African countries; yet, SCD care has not proportionally reached patients in these regions, and the disease has received limited attention in the past. Addressing the burden of SCD in sub-Saharan Africa requires a holistic, collaborative approach to ensure solutions are both comprehensive – i.e., cover the entire continuum of care from early diagnosis to treatment – and sustainable – i.e., are co-created and co-owned with local partners and integrated into existing local systems to enable long-term independence without the need for continuous external support.

**Objective:**

We outline a set of recommendations for enhancing the provision of comprehensive healthcare for prevalent diseases in resource-constraint settings, gathered from the Novartis Africa SCD Program, that could serve as ‘blueprint’ for public-private partnerships to tackle global health priorities.

**Methods:**

The Novartis Africa SCD program was initiated with the aim to bridge access gaps to SCD care and provide comprehensive and innovative treatment solutions for SCD, especially in SSA where the disease burden is highest. The Program was first inaugurated in 2019 in Ghana through a public-private partnership with the Ministry of Health of the Government of Ghana, the Ghana Health Service, and the Sickle Cell Foundation of Ghana. Through engagement with these partners, as well as with support from other organizations with complementary competencies and resources, several targeted solutions were implemented to help strengthen the healthcare ecosystem to allow for comprehensive SCD management. Learnings from these interventions are highlighted as best practice consideration as a catalyst and to activate more public-private actors for this neglected global health issue.

**Findings and Conclusions:**

A solid understanding of the access barriers to comprehensive care has to be acquired by listening to and learning from patients, civil society, and local experts. Access barriers need to be addressed at multiple levels, i.e., by not only making medicines available and affordable, but also by strengthening healthcare systems, building capacity, and fostering local research and development. Partnerships across governmental, public, academic, non-profit, and private organizations are needed to secure political will, pool resources, gather expertise with understanding of the local context, and allow integration into all levels of existing local healthcare structures and the wider society.

## Introduction

Sickle cell disease (SCD), an inherited disorder of haemoglobin, is associated with largely preventable morbidity and premature death, often in childhood [1]. The burden of SCD is highest in Sub-Saharan Africa (SSA), where 64%–76% of all neonates with SCD worldwide are born [2, 3], and statistical models indicate a childhood mortality of 50%–90% among infants with SCD [4]. In contrast, in the US, children born with SCD, who account for less than 10% of all SCD-affected neonates globally [2, 4], have a 90% chance to survive to adulthood [5].

In 2010, SCD was recognized by the World Health Organization (WHO) as a public health priority [6]. Nevertheless, the disease has been largely absent from global and national health agendas, which could be worsened by the recent shift of healthcare funding towards acute issues such as the COVID-19 pandemic [7]. In August 2022, African health ministers launched a WHO-supported campaign aimed to raise awareness of SCD, bolster prevention (genetic counselling and testing), and improve access to care in Africa; the campaign also stressed the need for stronger collaborations and partnerships to address the rising healthcare needs associated with SCD in Africa [8].

## Comprehensive, multi-stakeholder approaches are needed to ensure sustainable solutions

Traditionally, global health issues have been addressed using ‘philanthropic’ approaches of donations or affordable drug pricing. But a focus on medication cost alone does not necessarily translate into improved patient access. This was shown by an analysis of Novartis Access, a program offering medicines for non-communicable diseases at one USD per treatment, per month to patients in low- and middle-income countries. Data collected in the first year of the program in Kenya demonstrated only a limited impact on the availability of medicines in patient households [9].

The findings from the Novartis Access program highlight that due to the complexities of healthcare systems, especially those in underserved communities, comprehensive, holistic, and evidence-based approaches are needed to improve access to healthcare. The aim of such approaches is to consider all aspects of disease management throughout the patient journey (i.e., from awareness and early diagnosis through to treatment and follow-up care), and target all levels of care (i.e., primary, secondary, and tertiary), while accommodating the unique aspects of local health systems [4]. Furthermore, it is important to secure commitment and political will at a local level to ensure solutions are sustainable, i.e., solutions can exist in the long-term without the need for continuous external support [10]. This means solutions should be co-created and co-owned with local partners and be integrated into existing local systems [4]. Tackling this challenge requires multi-stakeholder collaborations, with partnerships across public, governmental, private, and societal sectors, to mobilize and share knowledge, expertise, technologies, and financial resources [4, 11].

Here we describe as an example of a multi-stakeholder collaboration the Novartis Africa SCD program, and outline recommendations based on our experience and learnings that may inform future public-private partnerships addressing other global healthcare issues.

The Novartis Africa SCD program was initiated with the aim to bridge access gaps to SCD care and provide comprehensive and innovative treatment solutions for SCD, especially in SSA where the disease burden is highest. The program was launched in 2019 in Ghana as the pilot country. Partners of the program included the government (Ghana Ministry of Health), a governmental health agency (Ghana Health Service), Novartis, and – as the implementing partner – a non-profit organization (Sickle Cell Foundation of Ghana [SCFG]). Together, they executed a Memorandum of Understanding (MoU) outlining a shared vision to implement holistic and sustainable solutions for expanding access to SCD care. Partnership with the government, who actively advocated for the program, was a critical determinant for the successful launch of the program.

## Understanding the barriers that limit access to care

**Figure 1:**
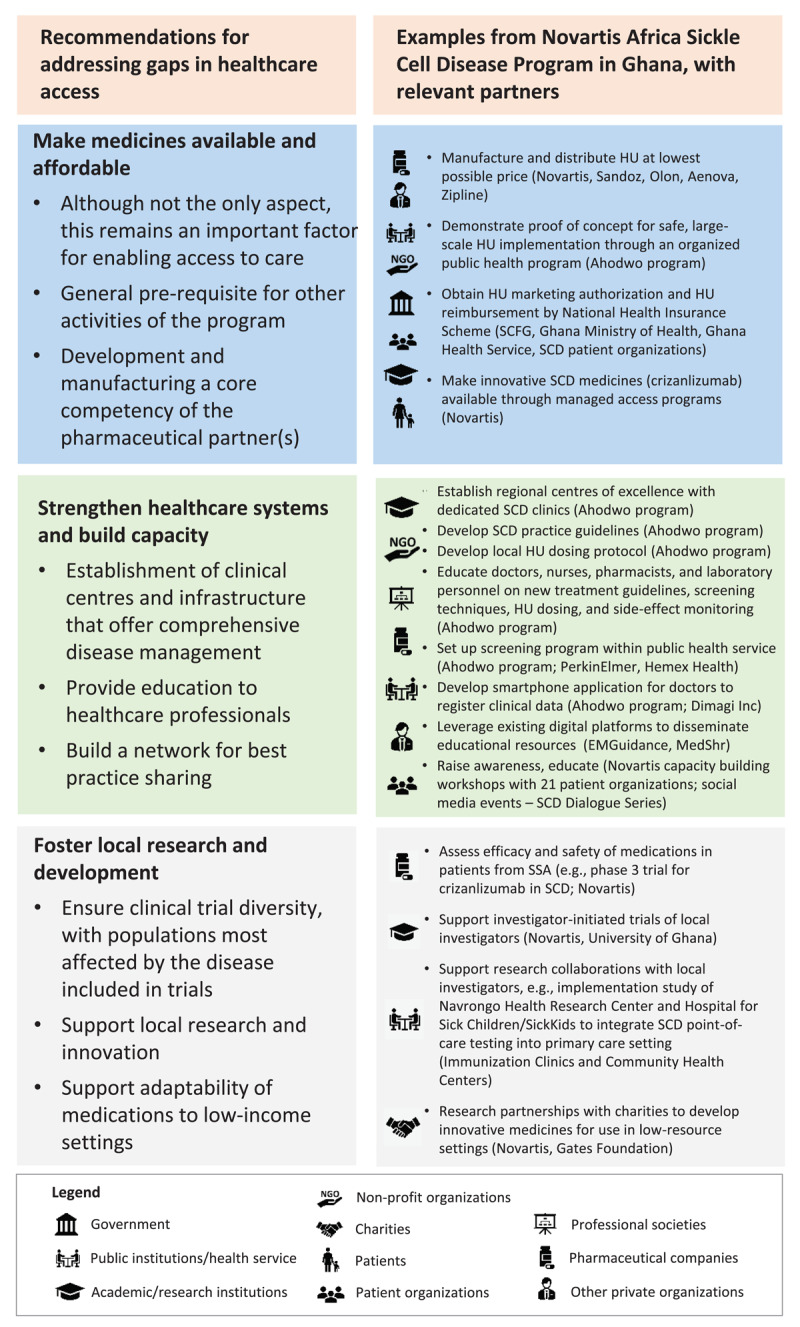
Recommendations for public-private partnerships for addressing access gaps, with examples from the Novartis Africa Sickle Cell Disease Program in Ghana. Ahodwo is a collaboration of the Sickle Cell Foundation Ghana, Ghana Ministry of Health, Ghana Health Service, and Novartis, and is led by a team of Ghanaian SCD experts. HU, hydroxyurea; SCD, sickle cell disease; SCFG, Sickle Cell Foundation Ghana.

As a pre-requisite to improving access to care in a comprehensive way, it is essential to gain a deeper understanding of all barriers and challenges that limit access. This can only be acquired by listening to patients, learning from civil society, and gathering insights from local experts to thoroughly understand the needs of all stakeholders in the healthcare system and along the entire patient journey [10]. In the case of the Novartis Africa SCD Program, some of the challenges observed with local stakeholders that limited access to SCD care are outlined in Table 1. To address these barriers in a comprehensive and sustainable way, we identified three main areas where collaborative partnerships can act (Figure 1).

**Table 1:** Challenges identified with local stakeholders that limit access to SCD care in Sub-Saharan Africa.


Limited availability and high cost of the first-line standard of care treatment for SCD (hydroxyurea)

Lack of a paediatric formulation of hydroxyurea

Lack of practice guidelines for the treatment of SCD

Lack of a national new-born SCD screening program

Limited knowledge among clinicians on how to manage SCD

Lack of facilities and treatment centres that offer comprehensive SCD care

Lack of electronic medical records

Limited awareness and prioritization of SCD among local governments and healthcare systems

Cultural stigma associated with being diagnosed with SCD


## Making medicines available and affordable

The first area is to make medicines available and affordable. Although this is not the only aspect of improving access to care, it remains an important factor as it is often a pre-requisite for other activities. In the case of the Novartis Africa SCD Program, this meant supporting the public availability and affordability of hydroxyurea, the generic, oral, first-line standard of care for SCD, which prior to the program was not widely obtainable in Ghana and most of SSA. Specifically, it involved manufacturing hydroxyurea at the lowest possible price, developing a child-friendly formulation, and, importantly, providing proof-of-concept that hydroxyurea treatment can be safely implemented and monitored on a large scale within the public health service in Ghana. The latter was executed and demonstrated by the Ahodwo program, a collaboration of the SCFG, Ghana Ministry of Health, Ghana Health Service, and Novartis, and led by a team of Ghanaian SCD experts, which focuses on widescale utilization of hydroxyurea treatment across Ghana [12]. These activities, together with additional efforts of the SCFG, local SCD patient organizations, and other allied agencies to raise awareness and enable prioritization of SCD among local governments and healthcare systems, resulted in obtaining market authorization for hydroxyurea with an indication for SCD by the Ghana FDA (October 2018), and its inclusion in the National Health Insurance Authority (NHIA) Scheme for universal access in Ghana (June 2022).

## Strengthening healthcare systems and building capacity

A second, key area for improving access to care is to strengthen local health systems, as these are the fundamental tool for providing people with all facets of healthcare. Health system strengthening involves activities such as building capacity of clinical centres and infrastructure to offer comprehensive disease management, providing education to healthcare professionals, and establishing a network for sharing best practices. Examples from the Novartis Africa SCD program, largely carried out by the Ahodwo program with support from other organizations with complementary competencies and catalytic investments from Novartis, included setting-up of regional treatment centres across Ghana for safe, large-scale implementation of hydroxyurea treatment, advocating for a national new-born screening program (collaboration with PerkinElmer and Hemex Health), disseminating national SCD practice guidelines, developing a smartphone application (called Meba-Nanti) to replace paper-based collection of patient data (i.e. new-born screening results, collaboration with Dimagi Inc), and leveraging existing digital platforms to disseminate educational materials (e.g., EMGuidance and MedShr). Another aspect of capacity building was to partner with local, regional, and global patient association groups (e.g., Global Alliance of Sickle Cell Disease Organizations) to raise SCD awareness, reduce stigma, and provide patient support, e.g., through patient information materials and social media campaigns.

## Fostering local research and development

A third area for improving access to care is to foster local research and development. This is important for clinical trial diversity to ensure patient populations most affected by the disease are included in new medicines research. Some of the key ongoing clinical trials assessing the efficacy and safety of Novartis SCD medicines in patients from SSA are a phase 1/2 trial for hydroxyurea (NCT01966731), and a phase 3 clinical trial for the new SCD treatment crizanlizumab (an anti-P-selectin monoclonal antibody; NCT03814746). Additionally, local industry-sponsored trials, support for investigator-initiated studies, and/or research collaborations are critical to further advance local clinical expertise, which may then also feed into strengthening of local health systems. For example, an implementation research collaboration between the Hospital for Sick Children (SickKids), the Navrongo Health Research Center, Novartis, and Hemex Health evaluates the feasibility of integrating point-of-care SCD testing into primary care centres (immunization clinics and community health centers) in Kassena-Nankana Districts in the Upper East Region of Ghana; if positive, SCD new-born screening could be extended to underserved rural communities in Northern Ghana and beyond.

## Conclusions and Outlook

Lessons learned from SCD, as well as other global diseases like malaria and HIV, have shown that integrated, holistic approaches are needed to address all facets of healthcare and involve stakeholders across all sectors, to help bridge access gaps in a sustainable way. Successful collaborations must continuously engage with all stakeholders and adapt as needed to respond to changing demographics, technologies, or ecosystems. For example, making medications affordable may allow patients to have treatment, but they may not be able to afford regular laboratory follow-up monitoring, clinics may run out of resources to cope with the increased demand for care, or the healthcare system may lack capacity for screening and diagnosing new patients.

It is important to evaluate the impact of global health interventions [9, 13]. In the case of the Novartis Africa SCD Program, there is an ongoing partnership with the University of Ghana to measure the impact of key aspects of the program such as large-scale implementation of hydroxyurea treatment within existing public health institutions. From early 2020 to mid-2022, the number of registered patients receiving hydroxyurea treatment in Ghana has increased by over 500% and is expected to rise further with the inclusion of hydroxyurea in the NHIA. The Meba-Nanti application is used by 24 facilities and currently holds screening data of over 65,000 new-borns. Within the first two years of the program’s implementation in 2019, the number of Program treatment centres in Ghana increased from 11 to 21. Additional agreements have been signed with other African governments in Kenya, Uganda, Tanzania, Zambia, and Angola. In 2022, the program also partnered with the American Society of Hematology’s Consortium on New-born Screening in Africa (CONSA) to provide seven African nations (Ghana, Kenya, Liberia, Nigeria, Uganda, Tanzania, and Zambia) with standard-of-care practices for screening and early intervention therapies for SCD, as well as to disseminate in these regions knowledge on disease burden and care through publications on research findings and professional education. A recent partnership between Novartis and the Bill & Melinda Gates Foundation aims to develop an accessible, single-administration, potentially curative *in vivo*-gene therapy for SCD in low-resource settings.

Industry support of global health programs could be perceived as a hidden promotional activity and an effort to ‘colonize’ global health [14]. By nature, pharmaceutical companies are profit-making organizations. Yet, the commitment to help improve access to care – including both standard of care and innovative therapies in an area where the pharmaceutical company has expertise and resources, and for those who need it most – can co-exist with the goal of being a profitable enterprise. In fact, it should be a business mandate to systematically integrate access strategies into the entire pharmaceutical research and development chain to globally share the responsibility of bridging gaps in healthcare, and it is also what society expects from the pharmaceutical industry.

Despite the efforts described here for the Novartis Africa SCD Program, there is still a long way to go to provide patients in SSA and other low-income countries with comprehensive SCD care. Additional global multi-sector partnerships are needed to complement and further the current efforts. In addition, including SCD in initiatives for non-communicable diseases would help put and maintain SCD on the agenda of health ministries in SSA, and give this neglected disorder the attention it needs. Beyond access to care for SCD, further partnership programs are needed for other global healthcare issues to help bridge the divide between those with access to critical healthcare and those without. Efforts will be maximally successful if they cover a multitude of sectors, leverage the core competencies of each partner, and are led by governments and other local stakeholders.
